# Pandemic Performance: Women Leaders in the COVID-19 Crisis

**DOI:** 10.1017/S1743923X20000549

**Published:** 2020-12

**Authors:** Andrea S. Aldrich, Nicholas J. Lotito

**Affiliations:** 1 Yale University; 2 Yale University

**Keywords:** COVID-19, pandemic, leaders, women

## Abstract

Media outlets have reported that women leaders around the globe are managing the COVID-19 crisis better than their male counterparts, responding faster and communicating better about pandemic policies. In this article, we examine empirical data on the timing of policy responses from the Coronavirus Government Response Tracker to determine whether and how countries led by women reacted differently to the pandemic. Exploring the relationship between the gender of leaders and legislators and the timing of stay-at-home orders, school closures, and coordinated public information campaigns, we find no statistical evidence supporting popular claims in the media. However, we find some evidence that the level of gender equality in legislatures is related to school closures, a policy with clear gendered consequences. These conclusions are an important first step in understanding the potentially gendered nature of the crisis response and identifying new avenues for research.

Many media sources are reporting that woman leaders around the world are “performing better” than men in handling the coronavirus disease 2019 (COVID-19) pandemic. This article examines these claims by exploring patterns in policy responses across the globe and connecting those patterns to the gender of political leadership. We investigate whether countries led by women are, in fact, reacting differently than those led by men, especially in the areas of pandemic control and containment and public health information, by analyzing data on COVID-19 policy responses provided by the Coronavirus Government Response Tracker (Hale et al. 2020) combined with data on the gender of government leaders and the percentage of women in legislatures. We find no reliable evidence of policy differences across women and men in response to the pandemic. However, the share of women in legislatures is associated with a delay in closing schools, consistent with women policy makers placing a higher social and economic value on schools remaining open. While our results fail to support claims that women leaders have responded more competently to the COVID-19 pandemic, they suggest that differences in women's and men's policy preferences may result in divergent policy outcomes.

## WOMEN AS LEADERS

Many of the sources making claims about the success of women leaders argue that women leaders were quicker to restrict the movement of citizens *(New York Times* 2020) or simply better at communicating both the seriousness of the virus and the responsibility of citizens to adhere to pandemic protocols (Henley and Roy 2020). However, few media sources mention that many of these women come from highly developed, rich democracies. Thus, it is important to identify patterns in policy responses that may vary by the gender composition of the government in order to both understand leadership style in the pandemic and identify potential avenues of research.

While media accounts rely mainly on anecdotes, prior research gives us reason to think that women leaders might make choices differently than men in times of crisis. For example, the decision to restrict movement and essentially close economies was undoubtedly one that weighed the risk of viral spread against disruptions to society and the economy. Women are often considered more risk averse than men (Byrnes, Miller, and Schafer 1999), especially under stress (Mather and Lighthall 2012) or when making political decisions (Verge, Guinjoan, and Rodon 2015), which may make them less willing to accept health risks and more willing to act quickly.

In addition, a larger share of women in legislatures might be associated with the adoption of different solutions. For example, more women in the legislature has been associated with an increase in public health spending (Clayton and Zetterberg 2018). More inclusive institutions bring people to office with different experiences, perspectives, and understandings of potential policy impacts. The number of women in the legislature may also be a signal of overall gender equality in society. Including more women in the legislature expands the pool of candidates from which women leaders can be drawn and increases the likelihood that women will receive influential cabinet positions (Krook and O'Brien 2012). Thus, the share of women in legislatures can serve as a proxy for the level of power women hold in the government.

It is also possible that this particular crisis, an unprecedented global pandemic, is a unique case to which our previous theories may not apply. With so much uncertainty about the future of the virus and its long-term economic, social, and political effects, we naturally seek tangible explanations for more desirable outcomes. Because the women leaders highlighted in the media, such as Angela Merkel of Germany and Jacinda Ardern of New Zealand, are so pathbreaking in their own right, it is easy to see why they make good examples of leadership excellence in these uncertain times.

However, given the paucity of women executives, these claims may be related to other characteristics shared by countries choosing women leaders. The likelihood that a woman reaches executive office is highly contingent on institutions both within the government and within the leadership selection process (Jalalzai 2013). Winning often involves playing by men's rules, and thus the winning women are much like other male politicians (Schwindt-Bayer 2011). If this is the case, we could expect differences in performance during a crisis to be attributed to many other factors.

## WOMEN IN COVID-19 RESPONSE

A first step in understanding the role of women's leadership in the pandemic is examining whether and how governments led by women behave differently than those led by men. We explore this by analyzing the timing of policy adoption in three areas: mandatory stay-at-home orders, school closures, and coordinated public information campaigns. We view these policies as representative of the actions that some women leaders took that have been lauded in the media as particularly effective. These containment measures were eventually embraced by most of the world's governments, but with significant variation in timing.^1^ Because containment policies are more effective the earlier they are adopted, we consider time to adoption a good measure of response effectiveness.

Stay-at-home orders are the clearest example of good policy because at the outbreak of the pandemic, containing the spread of the virus was seen as paramount to controlling its impact (McNeil 2020), the *timing* varied cross-nationally, and these measures had potentially far-reaching consequences. All of this should have entered into leaders’ risk calculations during the pandemic. School closings follow a similar logic to stay-at-home orders, except that they may disproportionately affect women who are also caregivers. We also study the implementation of public information campaigns, due to the media's emphasis on communication in women's leadership styles and its importance promoting the public's adherence to stay-at-home orders. Our expectation is that if women really are governing differently than men during this pandemic, we should see significant differences in the rollout of each measure.

## DATA AND ANALYSIS

Our three dependent variables are taken from the Coronavirus Government Response Tracker (Hale et al. 2020). This data set provides daily measures of policy responses beginning on December 31, 2019, through June 19, 2020.^2^ We calculate the number of days from the first recorded case of the virus in each country to the dates (if any) on which stay-at-home orders were implemented, schools were ordered to close, and a public information campaign was implemented.

We construct a binary measure of leader gender and a measure of the proportion of women in the lower house of the legislature. We incorporate both measures to test whether female heads of government have a significant relationship with the implementation of policy and to control for the percentage of women in the legislature as a proxy for the broader inclusion of women in political roles. Other political factors that can affect both policy choices and the ability to implement policy quickly are the type of government system (parliamentary versus presidential) and the ideology of the governing party. Therefore, we include an indicator for parliamentary systems, in which prime ministers may be more constrained in unilateral action than presidents, and an indicator for left parties, which may be more likely to implement policies of government intervention.^3^ We also include time-invariant measures of the level of economic (gross domestic product per capita) and political (Polity) development in each state, which can impact the state's capacity to make and implement policy. We control for population density and cumulative cases to account for the intensity of viral spread. In states where viral spread is occurring or can occur quickly, there may be greater incentives to implement policy quickly. We model policy adoption using a Cox proportional-hazards regression model (Cox 1972).^4^ We expect policy adoption to exhibit spillover effects, so we control for the number of countries worldwide that previously adopted the policy.

## RESULTS

Regression results are reported in Table 1.^5^ We find no evidence that leader gender affects time to implementation for any of the containment policies under investigation. Similarly, we find no significant effect of women in the legislature on either stay-at-home orders or information campaigns. These indeterminate findings fail to support the widespread claim that women leaders responded more competently and effectively than men to the COVID-19 pandemic.

**Table 1.** Main results

*Risk of Policy Adoption* *(Hazard Ratios)*	*(1)* *Stay-at-Home Order*	*(2)* *School Closing*	*(3)* *Information Campaign*
Woman leader	0.725	1.436	1.083
	(−0.78)	(1.03)	(0.23)
% Women in legislature	0.609	0.0181∗∗∗	1.971
	(−0.48)	(−4.24)	(0.81)
Total cases	1.393∗∗∗ (5.03)	1.690∗∗∗ (6.90)	1.170+ (1.83)
Global adoption	1.007+ (1.92)	1.014∗∗∗ (5.60)	1.006∗ (2.52)
Polity score	1.028	1.045∗	1.066∗∗
	(1.30)	(2.23)	(2.98)
GDP per capita	0.898 (−0.89)	0.931 (−0.54)	0.889 (−0.99)
Population density	1.031	0.985	1.095
	(0.37)	(−0.22)	(1.18)
Parliamentary system	1.377	0.842	1.443
	(1.30)	(−0.72)	(1.64)
Left government	1.750+	1.563	0.811
	(1.73)	(1.47)	(−0.74)
Observations	5,741	2,061	1,327
Countries	132	132	132

*Note:* Exponentiated coefficients; *t* statistics in parentheses.

+ *p* < .10; * *p* < .05; ** *p* < .01; *** *p* < .001.

However, we do find a robust *negative* relationship between women in the legislature and school closings. For each percentage increase in the proportion of women in the legislature, the risk of school closure falls by 0.98%. This effect suggests that countries where women are more involved in the policy-making process may produce different policy outcomes, perhaps reflecting a generally higher level of gender equality in society. Thus, governments with more women at all levels may be more aware of the social and economic costs of closing schools, such as the potential harm to children's development or the gendered impact of the loss of child care.

Because the number of women leaders is small, it is possible that any relationship between leaders’ gender and policy response is not detectable in a statistical framework. Therefore, we supplement the analysis with an exploration of sample means between countries with male and female heads of government, with the proviso that any observed differences are not statistically significant and could result from sampling variability.

When comparing sample means, countries with female leaders issued stay-at-home orders one day earlier than those with male leaders (22.6 days versus 23.6 days). However, countries with a higher share of women leaders, 31%, chose to eschew stay-at-home orders altogether, versus only 23% of men. By contrast, virtually every country eventually closed its schools, yet the average time to closure was four days longer in countries with women leaders (15.7 days versus 11.7 days). While this difference could be caused by random chance, it is consistent with our finding that countries with more women in the legislature were more likely to delay school closings. Finally, about half of all countries, and 63% of women-led countries, launched coordinated information campaigns before their first confirmed case of COVID-19. Among the remainder, time to implementation was one week shorter on average in countries with women leaders.

## CONCLUSION

Examining data on government response to the COVID-19 pandemic and its relationship to the gender of government leaders is an important first step in understanding the relationship between gender, leadership, and pandemic response. We offer one way in which we can begin to explore this relationship by looking at the time to implementation of many “successful” policies. While we found little definitive statistical evidence that women are making different choices than men in terms of time stay-at-home orders or information campaigns, we have shown that the decisions related to school closings may vary based on the preferences across genders. The conclusions we present should encourage future research that takes a more nuanced approach to the role of gender in government performance. It should caution against making broad generalizations about women or men in leadership during this unprecedented time and highlight the continued need to investigate the gendered nature of the crisis and the complex policy choices made every day.

## Supplementary material

For supplementary material accompanying this paper visit https://doi.org/10.1017/S1743923X20000549. click here to view supplementary material

## Footnotes

1. Policy adoption rates: stay-at-home orders, 63%; school closings, 81%; information campaigns, 80%.

2. See Section A of the appendix in the supplementary materials online for a full description of the data and sources. The data were collected by a team of country experts at the Blavatnik School of Government at the University of Oxford and made available to the public. We exclude China, where the virus originated, from our data.

3. Data on world leaders and left parties is taken, in part, from the Database of Political Institutions (Scartascini, Cruz, and Keefer 2018) and updated through 2020 by the authors. See Section A of the appendix for more information.

4. See Section B of the appendix for research design details and Section C for replication with a discrete-time model.

5. The exponentiated coefficients represent the hazard ratios for each predictor, such that a value over 1 indicates an increased “risk” of policy adoption and thus a lower average time to adoption.
