# Supplementary material for: Pandemic Performance: Women Leaders in the COVID-19 Crisis
Source: Politics & Gender. 2020 Dec;16(4):960–7. doi: 10.1017/S1743923X20000549 (PMC7853732; doi:10.1017/S1743923X20000549)
Supplement: Supplementary file 1 [file S1743923X20000549sup001.pdf]

Pandemic Performance: Women Leaders in the Covid-19 Crisis Online Appendix

Andrea S. Aldrich, Yale University

Nicholas J. Lotito, Yale University

A Description of the Data

The "Variation in government responses to Covid-19" data (Hale et al. 2020) team of country experts at the Blavatnik School of Government at the University of Oxford collected day to day policy information on government response to the pandemic in order to systematically track government response as the pandemic unfolded. This information was compiled into several index measures and made available to the broader community. The in-country data was collected by a team of country experts using publicly available data. From this data, we use the following three measures

- Stay at home requirements (C6): we recoded this as a binary, daily measure to capture the day in which each country required not leaving the house with only a few exceptions (2), requiring not leaving the house with only minimal exceptions (3). This ordinal variable is coded as follows in the original data:
  - No measures=0
  - recommend not leaving house=1
  - Require not leave house with exceptions for daily exercise, grocery shopping, and essential trips=2
  - Require not leaving house with minimal exceptions (allowances for once very few days, only on person at at time, etc.)=3
- School Closing (C1): scored as a ordinal measure in original data as listed below. We recoded this as a binary measure to capture the day in which each country fit the Criteria for requiring closing schools at some levels (2). This ordinal variable is coded as follows in the

original data:

- No measure=0
- Recommended closing=1
- Require closing (only some levels or categories)=2
- Require all closing all levels=3

- Public information campaigns (H1): we recoded this a binary measure to capture the day in which each country adopted a coordinated public information campaign (2) from the following ordinal measure:
  - No Covid-19 public information campaign=0
  - Public officials urging caution about Covid-19=1
  - Coordinated public information campaign (e.g. across traditional and social media)=2

Female leadership data was taken from the *CIA Chiefs of State and Cabinet Members of Foreign Governments* website (link) on May 11, 2020 and updated as necessary. We coded women who were heads of government. There are 16 female heads of government, which is only 10% of our sample. With the exception of Namibia (Prime Minister) and Myanmar (State Counsellor), where these positions are part of an executive presidency, this is head of government. Those countries are reported in Table 1.

Data on parliamentary and presidential systems if taken from the *Database of Political Institutions* (DPI). The variable *Parliamentary System* is recoded from the *System* variable to be 1 for governments with parliaments or assembly elected presidents and 0 for presidential systems. We also use this data to inform our left party coding, taking their guidelines for the coding of *EXECLRC* so that left parties are parties that identify at least in part as left wing or far left. We do not code centre-left parties as left. The DPI data was coded through 2017 and thus left many missing observations for new governments and since it relied on economic position data, was not entirely suitable for our use. Therefore we updated the data using a list of ruling parties available here and

Table 1: Women Government Leaders

Country	Name	Title
Aruba	Evelyn Wever-Croes	Prime Minister
Bangladesh	Shiekh Hasina	Prime Minister
Barbados	Mia Mottely	Prime Minister
Belgium	Sophie Wilmès	Prime Minister
Bolivia	Jaenine Áñez	President
Denmark	Mette Frederiksen	Prime Minister
Finland	Sanna Marin	Prime Minister
Germany	Angela Merkel	Chancellor
Iceland	Katrín Jakobsdótter	Prime Minister
Myanmar	Aung San Suu Kyi	State Counsellor
Namibia	Saara Kuungongelwa	Prime Minister
New Zealand	Jacinda Ardern	Prime Minister
Norway	Erna Solberg	Prime Minister
Serbia	Ana Brnabić	Prime Minister
Taiwan	Tsai Ing-wen	President

determined party family by various party websites. A complete list of ruling parties and their party families is not included in the replication files but is available upon request from the authors.

The percentage of women in the legislature was collection from the Inter-Parliamentary Union with a few exceptions. The following countries were missing from this data and were collected using the following sources:

- Aruba: (Hoey 2018)
- Kosovo: https://eca.unwomen.org/en/news/stories/2019/09/kosovo-women-parliamentarians-promote-younger-women-in-decision-making-and-politics
- Moldova: https://www.md.undp.org/content/moldova/en/home/blog/2019/cat-de-aproape-este-moldova-de-a-atinge-egalitatea-de-gen-.html
- Palestine: https://palestine.unwomen.org/en/what-we-do/leadership-and-political-participation/facts-and-figures#_ftn3
- Taiwan: (Sui 2016)

The national characteristics in the data are from a companion data set provided by the Oxford *Our World in Data* website. To these variables, we add a democracy score from the Polity5 project (Marshall and Gurr 2018) and GDP per capita and population density from the World Bank Development Indicators: https://databank.worldbank.org/.

B Research Design

Theoretically, we want to observe whether women leaders implement *better* policies in response to Covid-19; however, it is difficult to determine which policies are optimal, even *ex post*. Because global policy responses to the pandemic were unprecedented, we focus only on policies that were widely believed to be effective at the time of possible implementation, but where the social and economic consequences of such policies required careful consideration. We focus primary attention on one outcome: mandatory stay home orders, popularly known as "lockdowns." This policy was the most widely discussed and debated of the common governmental responses to Covid-19, and possibly the most consequential. Importantly, while all Covid-19 policy responses have been subject to debate, the effectiveness of stay home orders was widely assumed throughout the crisis and has been confirmed in retrospective analyses (Fowler et al. 2020).

We contrast our approach with the primary alternative, which assesses the outcome of Covid-related deaths (Bosancianu et al. 2020; Garikipati and Kambhampati 2020). Total reported deaths is a reasonable and important explicandum, and we encourage further study of its causes . However, we question the use of total deaths as a proxy for the quality of policy response. While stay home orders have been widely embraced in the crisis, the effectiveness of many other policies is less certain, complicating the relationship between policies and outcomes. Instead, many unobserved factors likely contribute to the worsening of the crisis in some countries, reducing our confidence in the results of such an analysis.

To test our hypotheses against the data, we estimate Cox proportional-hazard regression models of the form:

$$h_i(t) = h_0(t) + \exp(\beta_1 x_{i1} + \beta_2 x_{i2} + \cdots + \beta_k x_{ik})$$

where h represents the log-hazard of adopting a given policy, t represents the time since the country's first reported case, and $x_1 \ldots x_k$ are covariates. The main predictor variables are whether the head of government is a woman and the percentage of women in the legislature. We estimate the model by maximum likelihood using Efron's method for ties.

Our approach induces left-censoring, but we prefer it for two reasons. First, the data on policy adoptions is incomplete prior to each country's first reported case, so extending our data could introduce bias due to uneven reporting of policy responses. Second, this approach better accounts for variation in each country's risk of viral spread, resulting in better model fit (see Table 5).

Note that in a survival analysis, units drop from the data once they "fail" (i.e., adopt the given policy). Therefore, the total number of observations will be lower when modeling policies that were adopted sooner, on average. This accounts for variability in the reported n in our results tables.

C Additional Tests

Because we expect Covid-19 response to depend on several national-level variables, our main statistical models include several covariates. As a robustness check, we also perform Cox regression on each outcome using only a single predictor, either woman leader or women in the legislature. This approach, reported in Table 2, confirms our main results.

Our analyses rely on data in country-day format. In the main analyses, we treat time as continuous. As a robustness check, we replicate the main analysis using a discrete-time survival model, estimated by logistic regression with random effects by country. Results are reported in Table 3. The discrete-time analysis is generally consistent with our main findings. Note that in the logistic regression framework, the estimated coefficients are not directly interpretable.

We also replicate the main results using alternative thresholds for our three outcome variables. For the two containment policies, we consider more stringent criteria, namely: only the most restrictive stay home orders (with "minimal exceptions;" C6 = 3); and school closings *at all levels* (including universities; C1 = 3). Because providing public information was so common, we use

the most stringent level of this variable (public information campaign) in the main analysis, but we rerun the model using a lower threshold here (public officials urging caution; H1 = 1). Conversely, stay home orders with minimal exceptions were uncommon (less than 20% of all countries ever implemented them), so we are unable to estimate the effect of a female leader on the timing of these orders. Otherwise, our results are robust to this specification, as reported in Table 4.

An alternative modeling strategy would treat all countries as "at risk" for policy adoption from the beginning of the Covid crisis (*e.g.,* January 1, 2020). Unfortunately, policy actions are not always recorded in our data prior to a country's first case, so many observations are lost from these models. The results, presented in Table 5, are generally consistent with our main findings; however, these models perform poorly relative to the main models, reducing our confidence in this approach.

Table 2: Main Outcomes without Covariates

**Risk of Policy Adoption**						
Hazard Ratios	Stay Home		School Closing		Info. Campaign	
Woman Leader	0.832		0.839		1.281	
	(−0.58)		(−0.67)		(0.94)	
% Women Legislature		0.952		0.171**		1.487
		(−0.07)		(−2.69)		(0.61)
Observations	7454	7160	2428	2332	1798	1696
Countries	171	166	171	166	171	166

Exponentiated coefficients; t statistics in parentheses

$^{+}$ $p < 0.10$, * $p < 0.05$, ** $p < 0.01$, *** $p < 0.001$

Table 3: Discrete-Time Model

**Risk of Policy Adoption**	(1) Stay Home	(2) School Closing	(3) Info. Campaign
Woman Leader	−0.739	1.266	1.035
	(−0.37)	(0.63)	(0.41)
% Women Legislature	1.004	−16.10**	2.636
	(0.21)	(−2.61)	(0.42)
Total Cases	1.756***	1.364***	1.337***
	(6.22)	(4.00)	(3.53)
Global Adoption	0.114***	0.0736***	0.120***
	(6.02)	(4.28)	(5.08)
Polity Score	0.212+	0.146	0.578***
	(1.96)	(1.34)	(4.60)
GDP Per Capita	−1.484*	−0.740	0.364
	(−2.48)	(−1.17)	(0.44)
Population Density	0.831*	0.189	1.097*
	(1.97)	(0.46)	(2.31)
Parliamentary System	−0.160	−1.701	3.310*
	(−0.13)	(−1.37)	(2.12)
Left Government	4.161**	1.481	−3.634+
	(2.67)	(0.90)	(−1.86)
Observations	5741	2061	1327
Countries	132	132	132

Constant terms omitted; *t* statistics in parentheses

+ $p < 0.10$, * $p < 0.05$, ** $p < 0.01$, *** $p < 0.001$

Table 4: Alternative Outcome Measures

**Risk of Policy Adoption** *Hazard Ratios*	(1) Stay Home	(2) School Closing	(3) Info. Campaign
Woman Leader	$3.29e-20$	0.905	1.083
	(.)	(−0.26)	(0.23)
% Women Legislature	0.552	0.0228^{***}	1.971
	(−0.30)	(−3.62)	(0.81)
Total Cases	1.301^{*}	1.333^{***}	1.170^{+}
	(1.99)	(4.43)	(1.83)
Global Adoption	0.981	1.011^{***}	1.006^{*}
	(−0.66)	(4.25)	(2.52)
Polity Score	1.071	1.053^{*}	1.066^{**}
	(1.55)	(2.28)	(2.98)
GDP Per Capita	0.905	1.046	0.889
	(−0.44)	(0.34)	(−0.99)
Population Density	0.948	0.967	1.095
	(−0.30)	(−0.45)	(1.18)
Parliamentary System	0.257^{*}	0.732	1.443
	(−2.48)	(−1.34)	(1.64)
Left Government	1.045	1.901^{*}	0.811
	(0.06)	(2.07)	(−0.74)
Observations	12262	2510	1327
Countries	132	132	132

Exponentiated coefficients; *t* statistics in parentheses

$^{+}$ $p < 0.10$, * $p < 0.05$, ** $p < 0.01$, *** $p < 0.001$

Table 5: Alternative Window from January 1

**Risk of Policy Adoption** *Hazard Ratios*	(1) Stay Home	(2) School Closing	(3) Info. Campaign
Woman Leader	0.865	1.396	2.164
	(−0.36)	(0.89)	(1.47)
% Women Legislature	1.272	0.0949^{*}	3.145
	(0.25)	(−2.25)	(0.86)
Total Cases	1.107	1.129	1.211^{+}
	(1.59)	(1.63)	(1.94)
Polity Score	1.041^{+}	1.030	1.121^{***}
	(1.91)	(1.43)	(3.37)
GDP Per Capita	0.832	0.917	0.665^{+}
	(−1.36)	(−0.65)	(−1.92)
Population Density	1.128	1.032	1.197
	(1.53)	(0.46)	(1.36)
Parliamentary System	0.972	0.650^{+}	2.669^{**}
	(−0.12)	(−1.72)	(2.65)
Left Government	1.286	1.534	1.253
	(0.77)	(1.35)	(0.54)
Observations	5703	2042	1254
Countries	129	113	59

Exponentiated coefficients; *t* statistics in parentheses

$^{+}$ $p < 0.10$, * $p < 0.05$, ** $p < 0.01$, *** $p < 0.001$

References

Bosancianu, Constantin Manuel, Kim Yi Dionne, Hanno Hilbig, Macartan Humphreys, Sampada Kc, Nils Lieber and Alex Scacco. 2020. Political and Social Correlates of Covid-19 Mortality. Preprint SocArXiv.

Fowler, James H., Seth J. Hill, Nick Obradovich and Remy Levin. 2020. The Effect of Stay-at-Home Orders on COVID-19 Cases and Fatalities in the United States. Technical report medRxiv.

Garikipati, Supriya and Uma Kambhampati. 2020. Leading the Fight Against the Pandemic: Does Gender 'Really' Matter? Technical report.

Hale, Thomas, Noam Angrist, Beatriz Kira, Anna Petherick, Tony Phillips and Samuel Webster. 2020. Variation in Government Responses to Covid-19 Version 5 April 29, 2020. Blavatnik School Working Paper BSG-WP-2020/032 Oxford University.

Hoey, J. Kelly. 2018. "Aruba: Where Women Lead." *The New York Times* .

Marshall, Monty G and Ted Robert Gurr. 2018. "Polity5 Project, Political Regime Characteristics and Transitions, 1800-2018.".
URL: *https://www.systemicpeace.org/polityproject.html*

Sui, Cindy. 2016. "The Place to Be a Woman in Politics." *BBC News* .
